# A Dietary Feedback System for the Delivery of Consistent Personalized Dietary Advice in the Web-Based Multicenter Food4Me Study

**DOI:** 10.2196/jmir.5620

**Published:** 2016-06-30

**Authors:** Hannah Forster, Marianne C Walsh, Clare B O'Donovan, Clara Woolhead, Caroline McGirr, E.J Daly, Richard O'Riordan, Carlos Celis-Morales, Rosalind Fallaize, Anna L Macready, Cyril F M Marsaux, Santiago Navas-Carretero, Rodrigo San-Cristobal, Silvia Kolossa, Kai Hartwig, Christina Mavrogianni, Lydia Tsirigoti, Christina P Lambrinou, Magdalena Godlewska, Agnieszka Surwiłło, Ingrid Merethe Fange Gjelstad, Christian A Drevon, Yannis Manios, Iwona Traczyk, J Alfredo Martinez, Wim H M Saris, Hannelore Daniel, Julie A Lovegrove, John C Mathers, Michael J Gibney, Eileen R Gibney, Lorraine Brennan

**Affiliations:** ^1^ UCD Institute of Food and Health University College Dublin Dublin Ireland; ^2^ Creme Global Trinity Technology and Enterprise Campus Grand Canal Quay Dublin Ireland; ^3^ Human Nutrition Research Centre Institute of Cellular Medicine Newcastle University Newcastle Upon Tyne United Kingdom; ^4^ Hugh Sinclair Unit of Human Nutrition and Institute for Cardiovascular and Metabolic Research University of Reading Reading United Kingdom; ^5^ Department of Human Biology NUTRIM, School for Nutrition and Translational Research in Metabolism Maastricht University Medical Centre Maastricht Netherlands; ^6^ Department of Nutrition, Food Science and Physiology Centre for Nutrition Research University of Navarra Pamplona Spain; ^7^ ZIEL Research Center of Nutrition and Food Sciences Biochemistry Unit Technische Universität München München Germany; ^8^ Department of Nutrition and Dietetics Harokopio University Athens Greece; ^9^ National Food & Nutrition Institute (IZZ) Warsaw Poland; ^10^ Department of Nutrition Institute of Basic Medical Sciences University of Oslo Oslo Norway

**Keywords:** dietary feedback, Web-based dietary assessment tool, Food4Me, dietary decision trees, personalized nutrition, human nutrition

## Abstract

**Background:**

Despite numerous healthy eating campaigns, the prevalence of diets high in saturated fatty acids, sugar, and salt and low in fiber, fruit, and vegetables remains high. With more people than ever accessing the Internet, Web-based dietary assessment instruments have the potential to promote healthier dietary behaviors via personalized dietary advice.

**Objective:**

The objectives of this study were to develop a dietary feedback system for the delivery of consistent personalized dietary advice in a multicenter study and to examine the impact of automating the advice system.

**Methods:**

The development of the dietary feedback system included 4 components: (1) designing a system for categorizing nutritional intakes; (2) creating a method for prioritizing 3 nutrient-related goals for subsequent targeted dietary advice; (3) constructing decision tree algorithms linking data on nutritional intake to feedback messages; and (4) developing personal feedback reports. The system was used manually by researchers to provide personalized nutrition advice based on dietary assessment to 369 participants during the Food4Me randomized controlled trial, with an automated version developed on completion of the study.

**Results:**

Saturated fatty acid, salt, and dietary fiber were most frequently selected as nutrient-related goals across the 7 centers. Average agreement between the manual and automated systems, in selecting 3 nutrient-related goals for personalized dietary advice across the centers, was highest for nutrient-related goals 1 and 2 and lower for goal 3, averaging at 92%, 87%, and 63%, respectively. Complete agreement between the 2 systems for feedback advice message selection averaged at 87% across the centers.

**Conclusions:**

The dietary feedback system was used to deliver personalized dietary advice within a multi-country study. Overall, there was good agreement between the manual and automated feedback systems, giving promise to the use of automated systems for personalizing dietary advice.

**Trial Registration:**

Clinicaltrials.gov NCT01530139; https://clinicaltrials.gov/ct2/show/NCT01530139 (Archived by WebCite at http://www.webcitation.org/6ht5Dgj8I)

## Introduction

Diets that are low in saturated fatty acids (SFAs), sugar, and salt and high in fruit, vegetables, and fiber are considered the healthy choice and have been shown to reduce the risk of noncommunicable diseases (NCD) [1-4]. However, despite numerous campaigns and policies to promote healthy eating, NCD burden has continued to rise, globally, over the past decade with increased contribution from nutrition-related risk factors [5]. As a result, there is the need for effective strategies to promote healthy dietary habits and to help consumers to achieve the necessary dietary changes.

Intensive inter-person counseling and interventions have been shown to improve dietary behaviors [6], although the potential feasibility and effectiveness of such methods across large populations is limited by both expense and accessibility [1,7]. Given the global increases in Internet availability, and the increasing utilization of the Internet as a method for delivering behavioral changes [8-11], the use of Web-based dietary assessment tools to provide personalized nutrition (PN) advice (based on dietary intake alone) at an individual level could provide a more cost-effective approach for improving dietary behaviors than current generic dietary advice and inter-person counseling [1,12-14].

Numerous studies have shown that personalized feedback advice is more effective than generic information for changing health behaviors including dietary intake [8,15-20]. Web-based dietary assessment instruments (eg, food frequency questionnaires—FFQs, 24-hour recalls, food diaries) can be the basis for the development of tools with the ability to provide users with personalized or tailored dietary feedback advice based on their self-reported dietary habits [12]. Such dietary feedback advice often illustrates the adequacy of the individual’s nutrient intakes in comparison with recommended intakes, intakes of peers of the same age or gender or previously attained intakes [7,16,19-21].

Although Web-based dietary assessment tools provide a potential starting point for delivering personalized dietary advice to large populations, they are usually 1-way systems that are designed to collect dietary intake data in a cost-effective manner. To limit measurement error and increase data completeness, Web-based dietary assessment tools can be preprogrammed with range and plausibility checks, probing questions and encoded to ensure all questions are answered [22]. Despite programming these check elements, it is essential that dietary assessment systems are coherent, comprehensive, structured, and clear, particularly when a consistent approach for composing and delivering dietary feedback advice is required. Algorithms can be developed to provide feedback in a systematic manner and have been applied in many clinical or health care domains to link personal data to knowledge systems with the aim of developing diagnoses or treatment options. In the same way, by comparing nutrient intakes with dietary recommendations, it is possible to develop algorithms that generate appropriate feedback messages, which can be stored in a message archive [23,24]. Algorithms can also be programmed into dietary assessment tools, facilitating the automatic generation of feedback advice [8]. Web-based dietary assessment tools, which generate automatic feedback advice, have the potential to improve dietary habits across large population groups, while minimizing researcher burden, reducing costs, and saving time.

The objectives of this paper were to report on the development of a Web-based dietary feedback system for the delivery of consistent personalized dietary advice in a multicenter study and to examine the impact of automating the feedback system on dietary advice delivery.

## Methods

### Development of the Feedback System

The dietary feedback system was developed, for manual use by researchers, for the delivery of personalized dietary advice in the pan-European Food4Me Proof-of-Principle (PoP) study. The Food4Me PoP study that aimed to deliver Web-based personalized dietary and physical activity (PA) advice was designed to emulate a real-life Internet-delivered, PN service [25]. As described in detail elsewhere, 1607 participants were randomized to 1 of the 4 groups receiving different levels of PN advice: Level 0 (control group) receiving conventional, non-PN advice; Level 1 PN advice based on dietary intake and PA data alone; Level 2 PN advice based on dietary intake, PA, and phenotypic data; Level 3 PN advice based on dietary intake, PA, phenotypic, and genotypic data [25]. The aim was to recruit a total of 1540 participants (220 participants per center) to allow for a potential 20% dropout rate, planned using a priori power calculation [25]. Power calculations were conducted in Minitab and based on glucose and omega-3 fatty acid concentrations within European adult populations [25]. The study had ethical approval from the corresponding committees of all participating centers (Germany, Greece, Ireland, the Netherlands, Poland, Spain, and the United Kingdom) and was conducted from August 2012 to March 2014. The Food4Me trial was registered as a randomized control trial (NCT01530139) at Clinicaltrials.gov.

**Figure 1:**
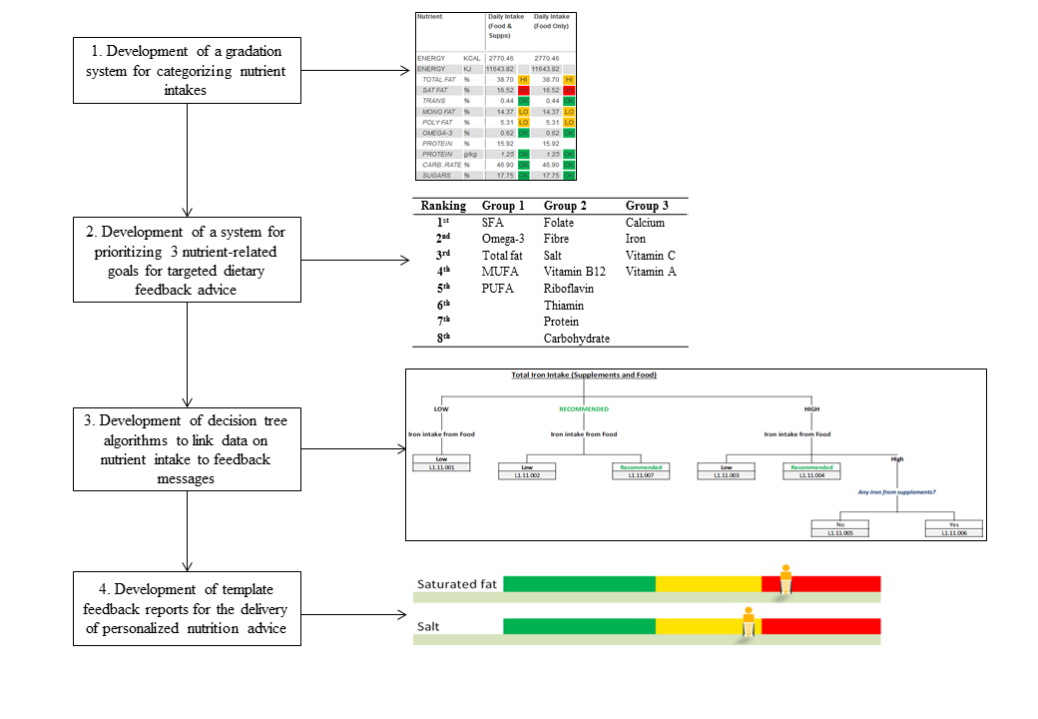
Stages in the development of the dietary feedback system.

The dietary feedback system, described in this paper, was used to deliver PN advice to participants randomized to receive Level 1 PN only (n=414). All participants of the Food4Me study received dietary feedback advice via email without face-to-face contact with researchers [25]. Thus, the feedback system was designed and developed to ensure that delivery of personalized dietary feedback was consistent across all the 7 countries. The key stages in the development of the dietary feedback system are illustrated in Figure 1 and described in detail in the following section.

#### Development of a Gradation System for Coding of Nutrient Intakes

Dietary intake data were collected throughout the Food4Me study using the recently developed and validated Web-based Food4Me FFQ [26,27]. Previous evidence has shown the Food4Me FFQ to have good agreement with the EPIC-Norfolk FFQ and moderate agreement with a 4-day weighed food record for the assessment of both nutrient and food group intakes, rendering it a useful tool for ranking individuals based on nutrient and food group intakes [26,27]. The Institute of Medicine (IOM) dietary reference intakes were used as a basis for developing a gradation system to categorize nutrient intakes automatically after completion of the Web-based Food4Me FFQ by participants [28,29]. Institute of Medicine reference values were used because (1) they were the most up-to-date values available and (2) recommendations vary across Europe with a need to find standardized reference intakes. Nutrient gradations were calculated based on IOM estimated average requirements (EARs) and tolerable upper intake levels (upper limits—ULs). Lower cutoff values were calculated as the EAR minus 2 standard deviations. Institute of Medicine–recommended daily allowance or World Health Organization recommendations were used to calculate the gradations when IOM EARs were not available [30,31]. The gradations (EAR, UL, and lower cutoff point) for each nutrient were preprogrammed into the Web-based Food4Me FFQ by Creme Global (Dublin, Ireland) facilitating a visual ranking system for nutrient intakes (Multimedia Appendix 1). Where applicable, gradations incorporated IOM recommendations for age and gender. Color coding and labeling of nutrient intakes were integrated into the Web-based Food4Me FFQ nutritional outputs to enable rapid visual assessment of the nutrient intake as *“very low,” “low,” “recommended,” “high,” *or* “very high,” * which were labeled and color-coded as red, amber, green, amber, and red, respectively.

#### Development of a System for Prioritizing Dietary Feedback Advice

 Seventeen nutrients were selected for inclusion in the dietary feedback system viz *“protein,” “carbohydrate,” “total fat,” “MUFA,” “PUFA,” “SFA,” “salt,” “omega 3,” “fiber,” “calcium,” “iron,” “vitamin A,” “folate,” “thiamin,” “riboflavin,” “vitamin B12,” and “vitamin C”*. In addition, based on patient-centered models for facilitating dietary change, which emphasize that patients should focus on only a few goals at a time [25], 3 nutrient-related goals (target nutrients) were selected for particular emphasis in the feedback report. To select these 3 nutrient-related goals for subsequent dietary advice, the 17 nutrients were split into 3 groups (Multimedia Appendix 2). Group 1 consisted of all the “fat-related” nutrients: SFA, omega-3, total fat, monounsaturated fatty acid, and polyunsaturated fatty acid (PUFA). Group 2 included folate, dietary fiber, salt, vitamin B12, riboflavin, thiamin, protein, and carbohydrate. Group 3 consisted of calcium, iron, vitamin C, and vitamin A. A ranking system was embedded in the methodology for identifying target nutrients, so that nutrients at the top of each group received highest priority (ie, nutrients of higher public health concern). Generally, the highest priority nutrient, flagged “red” from each group was chosen as the nutrient-related goal, if no “red” nutrients were available, those flagged “amber” were chosen. In cases when only 2 nutrients were flagged “red” or “amber,” a third ‘green’ (recommended) nutrient was given with a positive message to maintain nutrient intake.

#### Development of Decision Trees to Link Nutritional Intake Data to Feedback Messages

Sixteen dietary decision trees were manually developed to provide dietary feedback advice (Level 1 PN). All decision trees were developed to link nutrient intakes generated automatically by the Web-based Food4Me FFQ to a library of feedback messages. With the exception of the decision trees for carbohydrate, unsaturated fat, dietary fiber, and salt, all decision trees were generated to account for nutritional intake from both foods and supplements. Decision trees were based on the IOM gradation system with branches developed for “low,” “recommended,” and “high” intakes of each nutrient. The decision trees for SFA and salt were more complex and involved identifying the 2 main food groups contributing to the intake of the nutrient thereby enabling further personalization of dietary advice. For example, if a participant’s diet were identified as high in SFAs, researchers would follow the decision tree to identify the 2 highest contributing food groups of 7 potential candidate groups.

**Figure 2:**
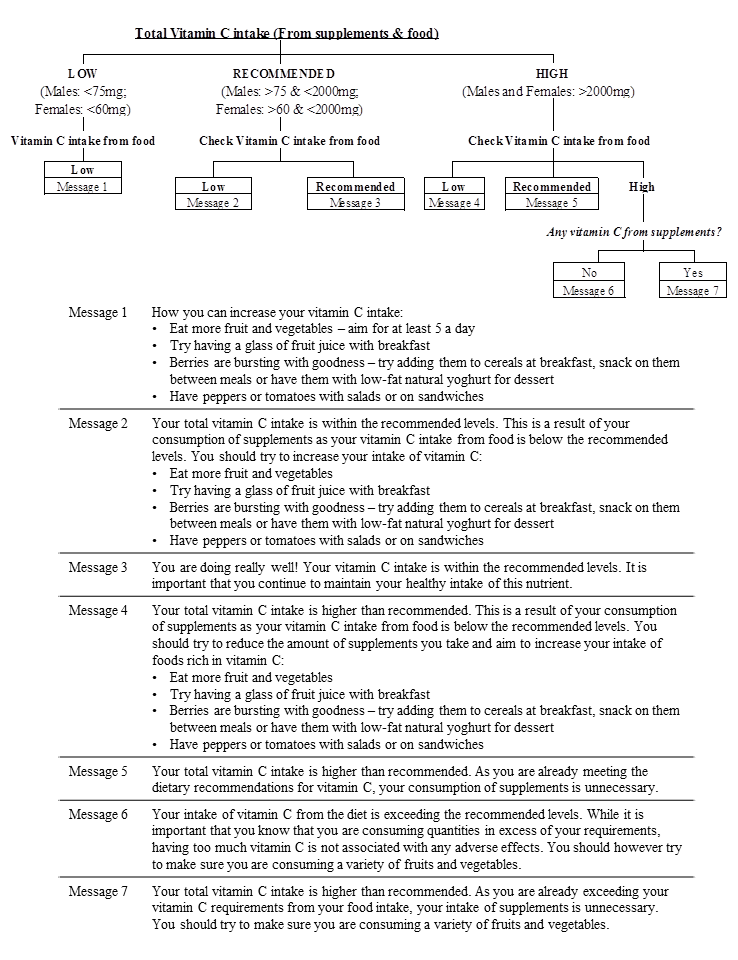
Vitamin C decision tree and corresponding feedback messages.

All decision trees included branches linking to individual feedback messages. In total, the archive of dietary feedback messages consisted of 92 messages, which were developed using a variety of reputable sources including British Dietetic Association, Food Safety Authority Ireland, and British Nutrition Foundation [32-36]. For each decision tree, the feedback messages consisted of practical food-based tips to improve nutrient intake. An example of the decision tree for vitamin C and corresponding feedback messages are presented in Figure 2, with further examples of SFA and salt given in Multimedia Appendix 3. Protocols for the feedback system were standardized across the 7 countries, and all feedback messages were translated into the language of each recruitment country. To assess the utility and applicability of the decision trees and corresponding feedback messages, during the Food4Me study, a record was kept by researchers at all the 7 centers. These entries were amalgamated and categorized by nutrient.

#### Development of Feedback Reports for Dietary Feedback Advice

Template feedback reports were developed to ensure that dietary advice was delivered in a consistent format across all countries. Standard operating procedures were compiled and nutritionists or dietitians in all centers received training in composing the reports. All feedback reports began with a short message of encouragement from the researcher, which also highlighted the main areas for improvement. This message was followed by 4 key sections:

- 1: How your diet compares to recommendations
- 2: Your physical characteristics
- 3: Your nutrient profile
- 4: Your personalized nutrition advice

Section 1 included a table comparing the participant’s average number of portions of 5 food groups: * “fruit and vegetables,” “whole grains,” “dairy products,” “oily fish,” *and *“red meat” * with guideline amounts. The guideline amounts were derived from amalgamating the national dietary advice in each of the 7 centers to create 1 common set of Food4Me dietary guidelines. Section 2 detailed the participant’s height and weight and compared the participant’s body mass index (BMI) and PA with recommendations. Section 3 graphically illustrated the participant’s intake of each of the 17 nutrients as “good, no change recommended,” “improvement recommended,” and “improvement strongly recommended” on a gradation scale comparing intakes with IOM recommendations. Section 4 detailed personalized behavioral goals including a table listing the participant’s 3 nutrient-related goals, along with dietary sources and the feedback message(s) from the corresponding decision tree. A list of all the food groups and nutrients for which personalized feedback was given can be found in Multimedia Appendix 4, with an example feedback report presented in Multimedia Appendix 5.

### Automation of the Dietary Feedback

Nutrient intake analysis was automated for use in the Food4Me study, with the nutrient-related goals and feedback messages derived manually (using the priority system and decision trees) by Food4Me researchers using their own judgment to overrule the feedback system when appropriate. After completion of the Food4Me intervention study, the feedback system was automated by Creme Global (Dublin, Ireland). Therefore, a comparison of the automated and manual-based approaches was possible. The system was automated by firstly capturing the manual decision process for selecting priority nutrients, based on nutrient intake analysis, in a computer algorithm (written in the PHP programming language). Second, the decision trees for selection of feedback messages were encoded in table-like data structures and stored in a relational database (MySQL). Additional computer algorithms for traversing these data structures were then developed (also using PHP), which enabled feedback messages to be generated automatically from nutrient intake analysis.

### Analysis

The first part of the analysis examines the manual use of the feedback system during the Food4Me PoP study. Descriptive statistics were computed to describe the general characteristics of participants across each of the 7 centers using general linear model analysis with least significant difference post hoc. Nutrient-related goal selection frequency was examined across each of the 7 countries. All statistical analyses were conducted using IBM SPSS Statistics version 20, and *P*<.05 was considered statistically significant.

**Figure 3:**
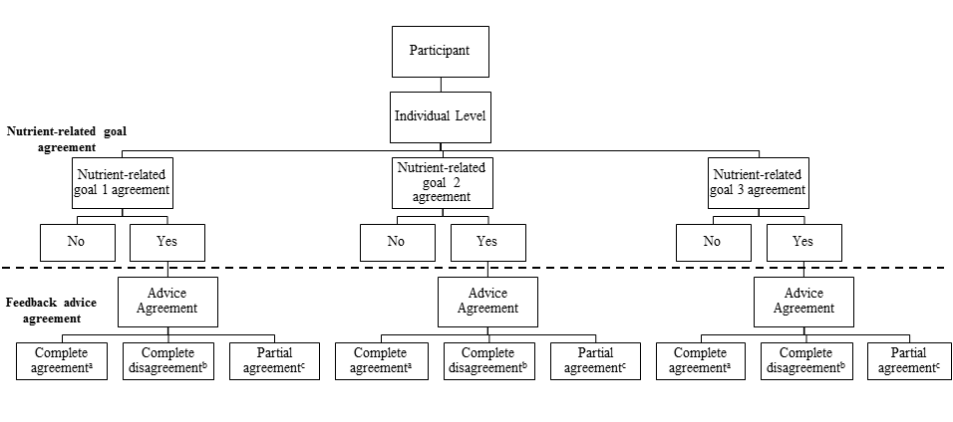
Steps taken to compare the level of agreement between the manual and automated systems for nutrient-related goals and feedback advice. The agreement between the 2 systems in selecting the 3 nutrient-related goals was assessed for each participant. Nutrient-related goal agreement between the 2 systems was also evaluated for all 3 nutrient-related goals in random order. Where there was agreement between the 2 systems for nutrient goal selection, agreement in the feedback advice message(s) selected was then assessed between the two systems. In Figure 3, a represents complete agreement, 100% match between advice messages given by the manual and automated systems; b represents complete disagreement, 0% match between the advice messages given by the 2 systems; c represents partial agreement, some agreement, and some disagreement between the advice messages given by the 2 systems, partial agreement was only applicable to the SFA and salt decision trees as multiple advice messages were given in the feedback to participants.

The second part of the analysis compares the dietary feedback provided by researchers during the Food4Me PoP study with feedback generated automatically by the computerized algorithms and messaging system for participants in Level 1 of the intervention. The level of agreement between the manual (researcher) and automated systems was assessed for both nutrient-related goal selection and feedback advice using baseline data for Level 1 participants, as illustrated in Figure 3. Agreement between the manual and automated systems, in selecting the 3 nutrient-related goals, was investigated by comparing each individual nutrient-related goal (1, 2, and 3) selected by both systems (manual vs automated) for each participant in the 7 countries. To evaluate the agreement for nutrient-related goal selection, we also examined whether the same 3 nutrients were selected as the 3 nutrient-related goals (in any order 1, 2, or 3) by both systems.

As outlined in Figure 3, to compare if the same feedback advice message(s) were derived from each decision tree by both systems, we selected only participants given the same nutrient-related goals (1, 2, and 3) by both the manual and automated systems and cross-compared the messages given by both systems for each nutrient-related goal (1, 2, and 3). Agreement was categorized as either “complete agreement,” “complete disagreement,” or “partial agreement” (data not shown). Overall agreement for feedback advice message was computed by summing the number of participants categorized into the 3 agreement groups for each nutrient-related goal across the countries.

## Results

### The Study Population

A total of 414 participants, from the Level 1 group, across the 7 countries were available for inclusion in the analysis. After removing dropouts immediately after being randomized (n=41) and participants who did not have nutrient-related goals recorded at baseline (n=4), 369 participants were included in the analysis (Germany, n=52; Greece, n=51; Ireland, n=55; Netherlands, n=56; Poland, n=49; Spain, n=54; and the United Kingdom, n=52). There were no significant differences in age for men and women; however, self-reported BMI was significantly lower for women compared with men (*P=*.002). When comparing demographic characteristics across all the countries, no significant differences were observed for bodyweight or BMI; however, significant differences were observed for age (*P=*.02) and height (*P<*.001), as summarized in Table 1.

**Table 1:** Demographic characteristics of the study population in total and across countries^a^.

Country	Demographic characteristics				
	Gender—female (%)	Age (y)	Height (m)	Weight (kg)	BMI (kg/m^2^)
All countries (n=369)	57.5	39.7 ± 12.9	1.71 ± 0.10	74.19 ± 16.61	25.21 ± 5.02
Germany (n=52)	51.9	42.6 ± 14.9^b,d^	1.75 ± 0.09^b^	73.48 ± 13.55	23.95 ± 3.62
Greece (n=51)	58.8	38.1 ± 10.5^b,c^	1.69 ± 0.10^c^	76.02 ± 19.37	26.46 ± 6.52
Ireland (n=55)	56.4	38.9 ± 12.0^b,c,d^	1.70 ± 0.10^c^	72.90 ± 16.13	25.07 ± 4.76
Netherlands (n=56)	50.0	43.0 ± 15.4^d^	1.76 ± 0.10^b^	77.70 ± 16.29	25.08 ± 4.37
Poland (n=49)	71.4	36.2 ± 11.1^c^	1.69 ± 0.07^c^	71.46 ± 16.61	24.83 ± 4.99
Spain (n=54)	51.9	41.8 ± 11.0^b,d,e^	1.69 ± 0.10^c^	74.83 ± 16.94	26.09 ± 4.98
United Kingdom (n=52)	63.5	37.1 ± 13.4^c,e^	1.70 ± 0.76^c^	72.60 ± 17.05	24.94 ± 5.36

^a^Values are means ± standard deviations.

^b,c,d,e^Means with different superscripts denote significant differences (analysis of variance with least significant difference post hoc)

### Manual Use and Efficacy of the Dietary Feedback System

The top 3 most frequently selected nutrient-related goals (1, 2, and 3) across each of the countries are summarized in Table 2. Saturated fatty acid was most frequently selected as nutrient-related goal 1 across all centers except for Spain where PUFA was most frequently selected as nutrient-related goal 1 in feedback given to 35% (19/54) of participants. Salt was most frequently selected as nutrient-related goal 2 in all 7 centers, with the exception of Germany, where both folate and salt were most frequently selected. As summarized in Table 2, greater variation was observed across the 7 countries for nutrient-related goal 3. As a result of the prioritization process, salt was most frequently selected as both nutrient-related goals 2 and 3, in both Spain and the United Kingdom.

The number of times each of the 17 nutrients was selected as a nutrient-related goal (1, 2, or 3) across each of the 7 countries is summarized in Table 3. Overall, SFA, salt, and dietary fiber were the top 3 most frequently targeted nutrient-related goals provided to 72%, 72%, and 39% of participants, respectively. Saturated fatty acid was the most frequently chosen nutrient-related goal overall for Germany, Ireland, and Poland. Salt was the most frequently selected nutrient-related goal overall for the Netherlands, Spain, and the United Kingdom, and in Greece, both dietary fiber and salt were most frequently selected.

A summary of the key issues encountered during the Food4Me study regarding the use and applicability of the decision trees and feedback advice messages is summarized in Table 4. Most of these issues were related to the SFA and salt decision trees, owing to their more complex design.

**Table 2:** Top 3 most frequently selected nutrient-related goals (1, 2, and 3) at baseline in the 7 countries^a^.

Country	Nutrient-related goal 1	Nutrient-related goal 2	Nutrient-related goal 3
Germany (n=52)	SFA^b^(54%)	Folate or salt (29%)	Carbohydrate or calcium (15%)
Greece (n=51)	SFA (41%)	Salt (39%)	Dietary fiber (41%)
Ireland (n=55)	SFA (56%)	Salt (53%)	Dietary fiber (15%)
Netherlands (n=56)	SFA (39%)	Salt (38%)	Dietary fiber 21%)
Poland (n=49)	SFA (53%)	Salt (39%)	Calcium (19%)
Spain (n=54)	PUFA^c^(35%)	Salt (35%)	Salt (24%)
United Kingdom (n=52)	SFA (48%)	Salt (46%)	Salt (21%)

^a^Percentages indicate the percentage of participants who received the nutrient as the nutrient-related goal.

^b^SFA: saturated fatty acid.

^c^PUFA: polyunsaturated fatty acid.

**Table 3:** Number of participants having nutrient-related goals (1, 2, or 3) at baseline across the 7 countries^a^.

Nutrient^a^	Germany (n=52)	Greece (n=51)	Ireland (n=55)	Netherlands (n=56)	Poland (n=49)	Spain (n=54)	United Kingdom (n=52)
Total fat	0	5	3	2	3	8	1
SFA^b^	42	27	47	43	38	30	37
MUFA^c^	6	2	3	13	11	3	12
PUFA^d^	6	16	8	3	3	21	13
Omega-3	8	6	2	6	0	2	4
Protein	2	0	2	4	2	0	1
Carbohydrate	9	1	4	2	2	1	3
Dietary fiber	14	31	20	19	14	24	20
Folate	23	23	8	13	23	20	10
Salt	31	31	46	47	31	35	45
Calcium	11	9	7	11	12	13	4
Iron	1	0	0	1	0	0	0
Vitamin C	2	1	3	2	4	2	1
Vitamin A	0	1	2	2	4	2	1
Vitamin B12	1	0	0	0	0	0	1
Total number of nutrient-related goals^e^	156	153	155	168	147	161^c^	153

^a^Riboflavin and thiamin were not given as nutrient-related goals in any of the 7 centers and are not presented in Table 3.

^b^SFA: saturated fatty acid.

^c^MUFA: monounsaturated fatty acid.

^d^PUFA: polyunsaturated fatty acid.

^e^Total number of nutrient-related goals in each country, calculated as number of participants (N) multiplied by 3. In Ireland, Spain, and the United Kingdom, total number of nutrient-related goals is less than this calculation as several participants were only given 2 nutrient-related goals in Ireland (n=10), Spain (n=1), the and United Kingdom (n=3).

**Table 4:** Issues with dietary decision trees or feedback messages.

Issue with decision trees or feedback messages	Decision trees affected
Algorithm did not capture all food groups contributing to the nutrient intake.	SFA^a^
Sometimes, the feedback messages are not specific to the participant’s diet, that is, the participant’s main sources of the nutrient may not have been identified in the feedback message.	SFA, salt, total fat
Feedback messages are repeated for different food groups.	Salt
Messages are not always relevant for vegetarians.	SFA, salt, omega 3

^a^SFA: saturated fatty acid.

### Comparison of Advice Generated by Manual and Automated Systems

The level of agreement between the manual and automated systems for the selection of the 3 nutrient-related goals in all the countries is summarized in Table 5. Good agreement was observed between both methods in all the countries for nutrient-related goals 1 and 2 with average agreement of 92% and 87%, respectively. Agreement between the 2 methods ranged from 100% (the United Kingdom) to 82% (Greece) for nutrient-related goal 1 and from 98% (Spain) to 80% (the Netherlands) for nutrient-related goal 2. Lower agreement was observed between the manual and automated systems for nutrient-related goal 3, mean 63% across centers. For nutrient-related goal 3 selection, agreement was highest for Spain (85%) and lowest for Greece (45%). Agreement between the 2 systems for all 3 nutrient-related goals in random order ranged from 83% (Spain) to 47% (Greece), averaging 66% across all countries.

Having selected the priority nutrients, the next stage was the selection of feedback messages. The level of agreement between the manual and automated systems for the feedback advice messages is summarized in Table 6. Complete agreement between the 2 systems for feedback advice ranged from 90% (Greece and Ireland) to 82% (Germany and Poland), with an average of 87% across the 7 countries.

**Table 5:** Level of agreement between manual and automated systems for baseline nutrient-related goals selection in all 7 countries^a^.

Country	Nutrient-related goal 1	Nutrient-related goal 2	Nutrient-related goal 3	Random order agreement^b^
	n (%)	n (%)	n (%)	n (%)
Germany (n=52)	50 (96)	47 (90)	33 (63)	36 (69)
Greece (n=51)	41 (82)	42 (82)	23 (45)	24 (47)
Ireland (n=55)^c^	48 (87)	45 (82)	31 (56)	33 (60)
Netherlands (n=56)	50 (89)	45 (80)	34 (61)	36 (64)
Poland (n=49)	44 (90)	43 (88)	35 (71)	37 (76)
Spain (n=54)^c^	53 (98)	53 (98)	46 (85)	45 (83)
United Kingdom (n=52)^c^	52 (100)	48 (92)	31 (60)	33 (63)
Average	(92)	(87)	(63)	(66)

^a^Level of agreement between the manual and automated systems for each individual nutrient-related goal and for all 3 nutrient-related goals in random order.

^b^Agreement for all 3 nutrient-related goals in random order derived by calculating the agreement when the same 3 nutrients were selected as the 3 nutrient-related goals by both systems regardless if they were given as nutrient-related goal 1, nutrient-related goal 2, or nutrient-related goal 3.

^c^Lower agreement observed for nutrient related-goal 3 and overall agreement as a minority some participants received only 2 nutrient-related goals from the manual system in Ireland (n=10), Spain (n=1), and the United Kingdom (n=3).

**Table 6:** Level of agreement between manual and automated systems for baseline feedback advice message selection in all 7 countries.

Country	Complete agreement^a^	Complete disagreement^b^	Agreement plus disagreement^c^
	n (%)	n (%)	n (%)
Germany (n=130)	107 (82)	3 (2)	20 (16)
Greece (n=107)	95 (90)	0 (0)	12 (11)
Ireland (n=124)	112 (90)	3 (3)	9 (7)
Netherlands (n=129)	114 (88)	0 (0)	15 (12)
Poland (n=122)	100 (82)	1 (1)	21 (17)
Spain (n=152)	134 (88)	5 (3)	13 (9)
United Kingdom (n=131)	113 (86)	3 (2)	15 (11)
Average	(87)	(2)	(12)

^a^Complete agreement, 100% match between advice messages given by the manual and automated systems.

^b^Complete disagreement, 0% match between the advice messages given by the 2 systems.

^c^Partial agreement, some agreement, and some disagreement between the advice messages given by the 2 systems, partial agreement was only applicable to the SFA and salt decision trees as multiple advice messages were given in the feedback to participants.

## Discussion

### Principal Findings

This paper presents a novel system for providing consistent personalized dietary advice, automatically generated from dietary intake data submitted via the Internet by participants in a multi-country study. To our knowledge, this paper is one of the first to describe, in detail, the steps involved in developing a dietary feedback system to translate food and nutrient intake data into automatically generated personalized feedback advice and to compare the automatically generated advice with advice manually provided by nutrition researchers.

Development of the feedback system consisted of a 4-step process: designing a gradation system to categorize nutrient intakes, creating a priority system to enable 3 nutrients to be further selected as nutrient-related goals for particular emphasis in the feedback report, constructing decision trees to link nutrient intakes to feedback advice, and finally, developing feedback report templates. The findings of this paper demonstrate that automation of the feedback system is feasible and more superior compared with manual use of the system by result of removal of human error.

The feedback system described in this paper is unique as it was developed to provide personalized feedback on intakes of 5 food groups and 17 nutrients, 3 of which were prioritized and selected as “nutrient-related goals” for subsequent targeted dietary advice. As the feedback system was developed to deliver relevant PN advice for all European adults, it was important that the system considered all nutrients for which changes in intake would be recommended to improve the health of significant proportions of the adult population. To address this requirement, the system that we developed included key food groups and a large number of nutrients so that feedback could be generated and personalized for a complete nutritional profile, rather than limited personalized information on one or several nutrient(s) as previously applied in other tailoring studies [20,37,38]. Providing extensive information, guaranteed that the dietary feedback would include personalized advice, which could be translated into food groups and nutrients of importance for a healthy diet (including fruit and vegetables, SFA, salt, fiber, whole grains, red meat and dairy products, omega-3 fatty acids, vitamin C, and iron) [39]. Several studies have briefly alluded to the application of a similar multistep process, linking nutrient intake data to feedback messages [1,13,21,24,37,40,41]. However, most of these studies have focused mostly on tailoring advice for selected nutrients or food groups only (eg, SFA, fruit and vegetable intakes) [1,6,13,16,21,24,37,42-44], with the exception of the advice generated for adolescents by Maes et al [40] which included fiber, vitamin C, calcium, iron, and fat and the reports developed by Kannan et al [23] that provided personalized feedback for 13 nutrients.

In addition to providing personalized food-based messages, feedback was also displayed graphically in reports by comparison of the individual’s nutrient intakes with dietary recommendations, similar to many other studies [1,6,15,16,42,45]. Nutrient-related goal selection was relatively consistent across the countries, with SFA and salt being the most frequently assigned nutrient-related goals targeted, although some North–South gradient differences are observed. This result was expected given the prevalence of diets high in SFAs and salt across Europe [39,46,47].

Because the feedback system had not been tested previously in a multicenter cohort, the decision trees were used manually for the delivery of dietary advice within the intervention study. Manual use of the decision trees within the study combined with rigorous recording of the issues identified by the nutritionists or dietitians delivering the intervention facilitated evaluation of the advice and identification of aspects of the system, which could be improved. The decision trees were developed to comprise essential components such as contribution from supplements and food groups (SFA and salt decision trees only). To guarantee feedback advice is fully relevant and appropriately personalized at an individual level, suggested improvements include further expanding the decision trees to integrate specific food items and incorporate additional food groups (SFA and salt decision trees), for example, branches for consumption of commonly consumed food items high in SFAs (eg, ice cream and quiche) could be added to the SFA and total fat decision trees.

Automated technologies are increasingly used across many disciplines and have been shown to be as effective as human (face-to-face) systems for 3-month weight loss and exercise interventions [8,48]. Furthermore, Emerencia et al [49] recently developed an automated personalized system for schizophrenia patients providing advice similar to that given by clinicians. Although several studies have generated computer-automated dietary feedback, to our knowledge, this paper is among the first to examine whether it is possible to fully automate a dietary feedback system designed to provide PN advice to enhance healthy eating. We observed high agreement between the manual and automated systems, averaging at 92% and 87%, for nutrient-related goals 1 and 2, respectively. Good agreement was also observed for feedback advice selection averaging 87%. However, although our results show potential for future automation of dietary feedback tools, some disagreement was observed between the manual and automated systems.

Nutrient goal selection disagreement averaged 8%, 13%, and 37% across the 7 centers for nutrient-related goals 1, 2, and 3, respectively. Reasons for this disagreement included: researchers overruling the priority system (eg, if they thought it was inappropriate to give one of the nutrient-related goals selected using the priority system); researchers misreporting the nutrient-related goal in the report (eg, the correct nutrient-related goal was given in the report but misreported in the Internet); and researcher error in selecting the nutrient-related goal (eg, giving an adequate nutrient by mistake). The level of disagreement between the 2 systems was much greater for nutrient-related goal 3, especially for Greece, primarily, a result of overruling the priority system. Overruling did not always mean that the original selection was inappropriate, only that the researcher thought another option was more relevant. For feedback advice selection, complete disagreement was negligible (2%), but partial agreement was higher (12%), and was confined to the SFA and salt decision tree messages only. Owing to their more complex design, the feedback advice for these nutrients included 2-4 individual messages, which were more prone to researcher misreporting, error or overruling the decision trees to give additional messages. There was also a degree of ambiguity in the prioritization rules and the SFA and salt decision tree rules. For example, in relation to prioritization, no rule was set for selecting a positive third nutrient when only 2 risk nutrients were identified, thereby increasing the level of disagreement for nutrient goal selection between the manual and automated systems. Similarly, for both the SFA and salt decision trees, no rules were established for which food groups should be selected when several contributed equally to the nutrient intake.

Although we observed some disagreement between the manual and automated systems, overall, the agreement between the systems was excellent. Much of the disagreement we observed between the 2 systems would be reduced by automation of the feedback system, which would eliminate researcher error and researcher misreporting, and further clarification of the decision rules for both the prioritization process and SFA and salt decision trees. Furthermore, despite researchers overruling the priority nutrients, both the systems selected nutrients of high priority.

Technological innovations have evolved the delivery and management of health care through the increasing use of mobile phone health and computerized health care apps [50,51], with the personalized health field developing rapidly [52]. Consequently, our feedback system has potential to become a cost-effective approach to improve dietary behaviors at an individual level, which could be delivered by public or private health care providers. The present system was designed to provide advice on usual intake rather than actual intake; the challenge in the future will be to provide advice to consumers at the point of purchase or consumption.

### Strengths and Limitations

The strengths of this study include the adequate sample size and multicenter recruitment in 7 European countries. In addition, to facilitate behavior change, the system was designed to incorporate numerous behavioral change techniques including goal setting, action planning, and barrier identification [11,25]. However, the system did not take into account motivation for change, and this could be a potential limitation. Feedback that is personalized to current dietary intake and stage of change may be considered as being more motivational and could have greater efficacy in promoting sustained dietary changes than advice personalized on dietary intake data only [14]. Another potential limitation of the study is that the feedback system was tested in a group of individuals who volunteered to receive PN advice and may therefore not be applicable to other population groups. However, the dietary and anthropometric characteristics of participants in the Food4Me study were broadly similar to those of the wider population of European adults so that the study tested the utility of the system to select appropriate feedback for the likely most common dietary changes. In addition, we did not follow-up with participants regarding their opinions about the advice they received, for example, if they thought it was relevant to them, appropriate, or useful, and this would have been extremely useful information to have captured for the future progression of PN.

### Conclusions

We developed a Web-based dietary feedback system that was capable of delivering consistent personalized dietary advice to adult European participants in the multicenter Food4Me study. Outcomes from comparison of the manual and automated feedback systems provide confidence that such an automated dietary feedback system can be developed and implemented across multiple countries with the potential to contribute to scalable and cost-effective interventions to improving dietary behaviors and health across large populations.

## Appendix

Multimedia Appendix 1 Nutrient gradations.

Multimedia Appendix 2 Level 1 priority nutrients and rationale.

Multimedia Appendix 3 Example decision trees and messages.

Multimedia Appendix 4 Nutrients and food groups for which personalized feedback was given.

Multimedia Appendix 5 Example feedback report.

## Abbreviations

BMI: body mass index
EAR: estimated average requirement
FFQ: food frequency questionnaire
IOM: Institute of Medicine
NCD: noncommunicable diseases
PA: physical activity
PN: personalized nutrition
PoP: Proof-of-Principle
PUFA: polyunsaturated fatty acids
SFA: saturated fatty acid
UL: upper limit
