# Supplementary material for: A Dietary Feedback System for the Delivery of Consistent Personalized Dietary Advice in the Web-Based Multicenter Food4Me Study
Source: J Med Internet Res. 2016 Jun 30;18(6):e150. doi: 10.2196/jmir.5620 (PMC4945818; doi:10.2196/jmir.5620)
Supplement: Multimedia Appendix 1 [file jmir_v18i6e150_app1.pdf]

Nutrient Gradations[a]

**Protein** (g/kgBW)	<0.52	>0.52 & <0.66	≥0.66 & ≤2.4	>2.4

**Carbohydrate** (%TE)	<40%	≥40 & <45%	45-65%	>65 & ≤70%	>70%

**Total Fat** (%TE)	<15%	≥15 & <20%	20-35%	>35 & ≤40%	>40%

**Monounsaturated** (%TE)	<10%	≥10 & <15%	15-20%	>20 & ≤30%	>30%

**Polyunsaturated** (%TE)	<5%	≥5 & <6%	6-11%	>11 & ≤12%	>12%

**Saturated Fat** (%TE)	<10%	≥10 &≤15%	>15

**Salt** (g)	18-50yrs	≤3.75	>3.75 & ≤5.75	>5.75
	51-70yrs	≤3.25	>3.25 & ≤5.75	>5.75
	>70yrs	<3	≥3 & <5.75	>5.75

**Omega-3** (%TE)	<0.2	≥0.2 & <0.6	≥0.6

**Fibre** (g) Males	18-50yrs	<28	≥28 & <38	≥38
	>50yrs	<20	≥20 & <30	≥30

**Fibre** (g) Females	18-50yrs	<15	≥15 & <25	≥25
	>50yrs	<14	≥14 & <21	≥21

**Calcium** (mg) Males	18-70yrs	<600	≥600 & <800	≥800 & ≤2500	>2500
	>70yrs	<800	≥800 & <1000	≥1000 & ≤2500	>2500

**Calcium** (mg) Females	18-50yrs	<600	≥600 & <800	≥800 & ≤2500	>2500
	>50yrs	<800	≥800 & <1000	≥1000 & ≤2500	>2500

**Iron**(mg) Males	>18yrs	<4	≥4 & <6	≥6 & ≤45	>45

**Iron**(mg) Females	18-50yrs	<3.15	≥3.15 & <8.1	≥8.1 & ≤45	>45
	>50yrs	<3.5	≥3.5 & <5	≥5 & ≤45	>45

**Vitamin A** (μg) Males	<350	≥350 & <625	≥625 & ≤3000	>3000

**Vitamin A** (μg) Females	<300	≥300 & <500	≥500& ≤3000	>3000

**Folate** (μg)	<240	≥240 & <320	≥320 & ≤1000	>1000

**Thiamin** (mg) Males	<0.8	≥0.8 & ≤1.0	>1.0

**Thiamin** (mg) Females	<0.7	≥0.7 & ≤0.9	>0.9

**Riboflavin** (mg) Males	<0.9	≥0.9 & ≤1.1	>1.1

**Riboflavin** (mg) Females	<0.7	≥0.7 & ≤0.9	>0.9

**Vitamin B12** (μg)	<1.6	≥1.6 & ≤2.0	>2.0

**Vitamin C** (mg) Males	<60	≥60 & <75	≥75 & ≤2000	>2000

**Vitamin C** (mg) Females	<45	≥45 & <60	≥60 & ≤2000	>2000

[a] MUFA, monounsaturated fatty acids; PUFA, polyunsaturated fatty acids; SFA, saturated fatty acids; TE, total energy. This table presents the gradations used to categorize nutrient intakes as "*very low*", "*low*", "*recommended*", "*high*" or "*very high*" automatically following completion of the online Food4Me food frequency questionnaire. Gradation cut-offs were calculated based on Institute of Medicine (IOM) estimated average requirements (EARs) and tolerable upper levels. Institute of Medicine recommended daily allowance or World Health Organisation recommendations were used when IOM EARs were not available.
