# Supplementary material for: A Dietary Feedback System for the Delivery of Consistent Personalized Dietary Advice in the Web-Based Multicenter Food4Me Study
Source: J Med Internet Res. 2016 Jun 30;18(6):e150. doi: 10.2196/jmir.5620 (PMC4945818; doi:10.2196/jmir.5620)
Supplement: Multimedia Appendix 2 [file jmir_v18i6e150_app2.pdf]

Level 1 priority nutrients and rationale[a]

Ranking	Group 1	Group 2	Group 3
1st	**SFA** [1,2] Ranked highest in this group as high SFA intake is a risk factor for CVD and other diseases. In Europe SFA intakes are generally above recommended.	**Folate** [4,5] Ranked highest in this group as folate intake is low in European countries.	**Calcium** [8,9,10,11] Ranked highest due to the incidence of osteoporosis in both males and females in the European population. Incidence of osteoporosis predicted to increase by 23% from 2010-2050.
2nd	**Omega-3** [3] Ranked second due to its health benefits in reducing disease and low intake in most European countries.	**Fibre** [6,7] Ranked second due to the low intakes of fibre in the European population and it has wider health implications than salt.	**Iron** [12,13,14,15] Ranked second due to the incidence of iron-deficiency anaemia in European countries. In Ireland 1 in 3 women have inadequate iron stores and 1 in 30 have iron-deficiency anaemia.
3rd	**Total Fat** [18] Ranked lower as SFA is of greater concern in the European population and if SFA is flagged then the advice will target total fat.	**Salt** [16] Ranked third as salt intakes are a concern in European countries.	**Vitamin C** [10] Lower ranking due to adequate intakes in the European population
4th	**MUFA** Ranked as lower priority, if SFA is flagged then the advice given will include changing SFAs to unsaturated fats.	**Vitamin B12** [17] Low priority due to adequate intakes in the European population	**Vitamin A** [17] Ranked as lower priority due to adequate intakes in the European population
5th	**PUFA** Ranked as lower priority due to the above fats having higher priority. If SFA is flagged then the advice given will include changing SFAs to unsaturated fats.	**Riboflavin** [17] Low priority due to adequate intakes in the European population. However greater proportion of adults with intakes less than the LRNI than thiamin, so ranked higher.	
6th		**Thiamin** [17] Low priority due to adequate intakes in the European population.	
7th		**Protein** [18,19] Low priority as protein is rarely a problem nutrient in the European population	
8th		**Carbohydrate** [7] Ranked as lower priority as carbohydrate intake is rarely a problem nutrient in the European population.	

[a]This table presents the rationale behind the priority nutrient system used to select the top 3 nutrient-related goals for targeted personalized feedback advice for Level 1 personalized nutrition. Generally nutrients which deviated most from the recommendations and were ranked highest in each of the priority systems were selected as nutrient-related goals.

References

1. Perk J, De Backer G, Gohlke H, Graham I, Reiner Z, Verschuren WM, Albus C, Benlian P, Boysen G, Cifkova R, et al. European guidelines on cardiovascular disease prevention in clinical practice (version 2012): the fifth joint task force of the European society of cardiology and other societies on cardiovascular disease prevention in clinical practice. Int J Behav Med; 2012;19:403-88. PMID: 23093473

2. Harrington KE, McGowan MJ, Kiely M, Robson PJ, Livingstone MB, Morrissey PA, Gibney MJ. Macronutrient intakes and food sources in the Irish adults: findings of the North/South Ireland Food Consumption Survey. Public Health Nutr ;2001;4:1051-60. PMID: 11820918

3. Von Schakey C, Harris WS. Cardiovascular benefits of omega-3 fatty acids. Cardiovasc Res 2007;73:310-5. PMID: 16979604

4. McNulty H, Scott JM. Intake and status of folate and related B-vitamins: considerations and challenges in achieving optimal status. BrJ Nutr 2008;99:S48-54. PMID: 18598588

5. Park JY, Nicolas G, Freisling H, Biessy C, Scalbert A, Romieu I, Chajès V, Chuang SC, Ericson U, Wallström P, et al. Comparison of standardised folate intake across ten countries participating in the European Prospective Investigation into Cancer and Nutrition. BrJ Nutr 2011:108:552-69. PMID: 22040523

6. Flynn MA, O'Brien CM, Faulkner G, Flynn CA, Gajownik M, Burke SJ. Revision of food based dietary guidelines for Ireland Phase 1: evaluation of Ireland's food guide. Public Health Nutr 2012;15:518-26. PMID: 21914255

7. Cust AE, Skilton MR, van Bakel MM, Halkjaer J, Olsen A, Agnoli C, Psaltopoulou T, Buurma E, Sonestedt E, Chirlaque MD, et al. Total dietary carbohydrate, sugar, starch and fibre intakes in the European Prospective Investigation into Cancer and Nutrition. Eur J Clin Nutr 2009;63:S37-60. PMID: 19888280

8. Cashman KD. Calcium intake, calcium bioavailability and bone health. Br J Nutr 2002;87:S169-77. PMID: 12088515

9. Roman Viñas B, Ribas Barba L, Ngo J, Gurinovic M, Novakovic R, Cavelaars A, de Groot LC, van't Veer P, Matthys C, Serra Majem L. Projected Prevalence of Inadequate Nutrient Intakes in Europe. Ann Nutr Metab 2011;59:84-95. PMID: 22142665

10. Kai MC, Anderson M, Lau EM. Exercise interventions: defusing the world's osteoporosis time bomb. Bull World Health Organ 2003;81:827-30. PMID: 14758410

11. Irish Osteoporosis Society. 2015. Patient guide to calcium and vitamin D3. http://www.irishosteoporosis.ie/images/uploads/Calcium%20%26%20Vit%20D%20July%202012%20Print%20Ready%20File.pdf. Archived at http://www.webcitation.org/6epGkYXcJ

12. Flynn A, Hirvonen T, Mensink GB, Ocké MC, Serra-Majem L, Stos K, Szponar L, Tetens I, Turrini A, Fletcher R, Wildemann T. Intake of selected nutrients from foods, from fortification and from supplements in various European countries. Food Nutr Res 2009;53:doi:10.3402/fnr.v53i0.2038. PMID: 20011225

13. Hannon EM, Kiely M, Harrington KE, Robson PJ, Strain JJ, Flynn A. The North/South Food Consumption Survey: mineral intakes in 18-64- year old adults. Public Health Nutr 2001;4:1081-8. PMID: 11820921

14. Hercberg S, Preziosi P, Galan P. Iron deficiency in Europe. Public Health Nutr 2001;4:537-45. PMID: 11683548

15. Bord Bia. 2014. Iron for women - the easy way. http://www.bordbia.ie/consumer/aboutfood/nutrition/Pages/Ironforwomen.aspx. Archived at http://www.webcitation.org/6epGPxWjO

16. European Union. 2010. European Commission Survey on Members States' Implementation of the EU Salt Reduction Framework. http://ec.europa.eu/health/nutrition_physical_activity/docs/salt_report1_en.pdf. Archived at http://www.webcitation.org/6epGWyW9Q

17. Mensink GB, Fletcher R, Gurinovic M, Huybrechts I, Lafay L, Serra-Majem L, Szponar L, Tetens I, Verkaik-Kloosterman J, Baka A, et al. Mapping low intake of micronutrients across Europe. Br J Nutr 2013;110:755-73. PMID: 23312136

18. Halkjær J, Olsen A, Overvad K, Jakobsen MU, Boeing H, Buijsse B, Palli D, Tognon G, Du H, van der A DL, et al. Intake of total, animal and plant protein and subsequent changes in weight or waist circumference in European men and women: the Diogenes project. Int J Obes (Lond) 2011;35:1104-13. PMID: 21139559

19. European Food Safety Authority. Scientific Opinion on Dietary Reference Values for protein. EFSA Journal 2012;10:2557. doi:10.2903/j.efsa.2012.2557.
