# Supplementary material for: A Dietary Feedback System for the Delivery of Consistent Personalized Dietary Advice in the Web-Based Multicenter Food4Me Study
Source: J Med Internet Res. 2016 Jun 30;18(6):e150. doi: 10.2196/jmir.5620 (PMC4945818; doi:10.2196/jmir.5620)
Supplement: Multimedia Appendix 3 [file jmir_v18i6e150_app3.pdf]

Saturated Fat Decision Tree

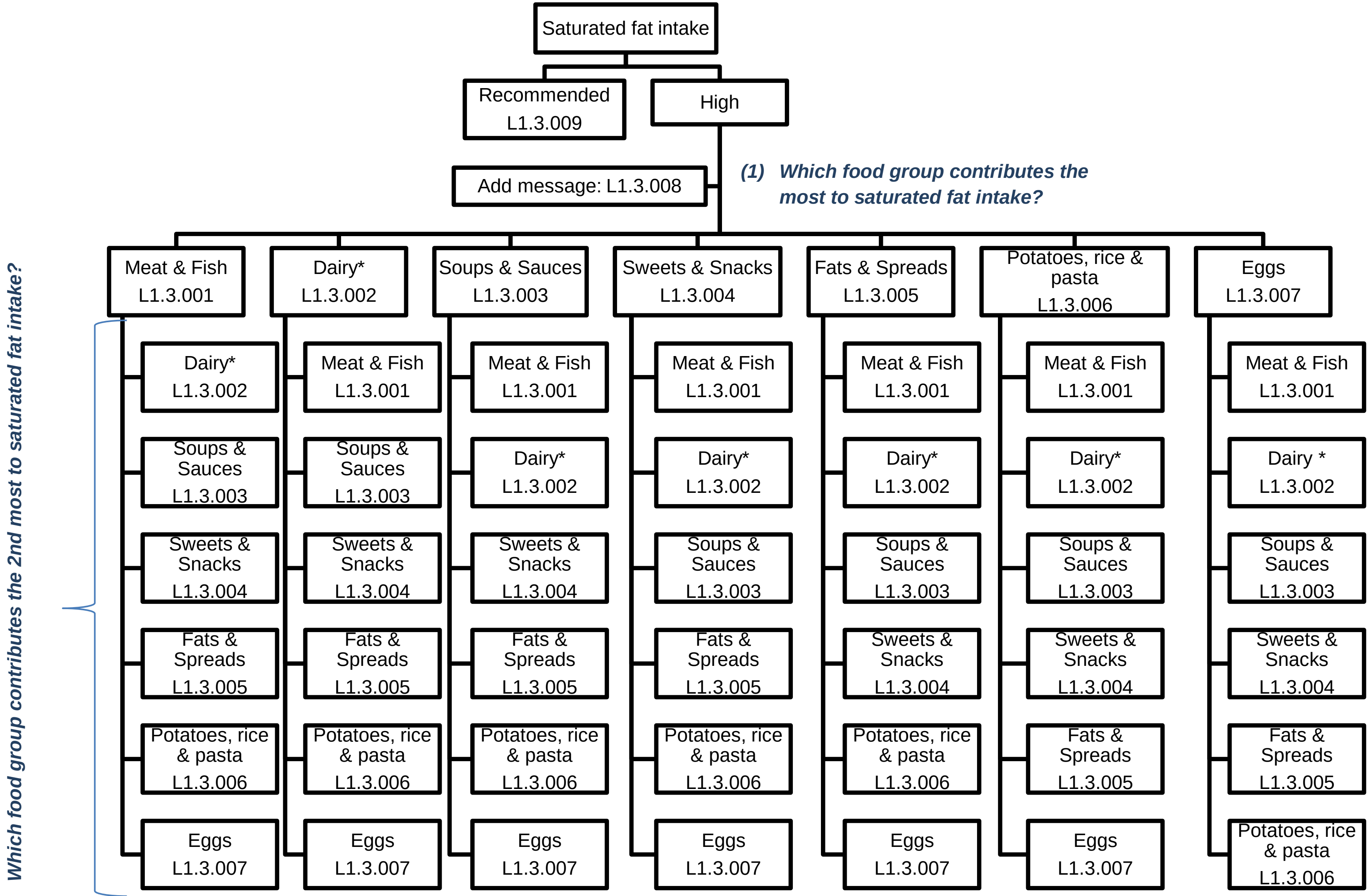

*If dairy is the highest or 2nd highest contributing food group then check which dairy food is the main contributor: if it is high fat cheese add message L1.3.002.1; if it is medium fat cheese add message L1.3.002.2; and if it is whole/full-fat milk add message L1.3.002.3

Saturated fat decision tree feedback messages

Message No.	Feedback message
L1.3.001	•Swap savoury pies and processed meats e.g. burgers, sausages and chicken goujons for lean meats or skinless chicken breast<br>•Trim the fat off meat before cooking it
L1.3.002	•Choose low-fat dairy products over full-fat dairy products and take care with your portion sizes
L1.3.002.1	•Swap high-fat cheeses for medium fat cheeses like Edam, goats cheese or camembert; even better try low-fat cheeses like feta, ricotta or cottage cheese
L1.3.002.2	•Go for low-fat cheeses like feta, cream cheese or cottage cheese
L1.3.002.3	•Swapping 1 pint of whole milk for 1 pint of skimmed milk can save you around 200kcal and 22g of fat!
L1.3.003	•Go for tomato based sauces and soups instead of creamy ones
L1.3.004	•Reduce your intake of cakes, biscuits and chocolates<br>•Go for treat-size bars and fill up on fruit, vegetables and unsalted nuts
L1.3.005	•Switch to use healthier unsaturated fats like sunflower, olive or rapeseed oil and low-fat spreads instead of butter<br>•Try to use smaller amounts of spreads and oils
L1.3.006	•Try to have readymade meals and takeaways only occasionally and watch your portion sizes<br>•For home cooking try to use low-fat ingredients i.e. reduced fat mayonnaise for potato salads or low-fat pasta sauces and cheeses for lasagne or pizza toppings
L1.3.007	•While eggs are an excellent source of many vitamins and minerals they also are a source of fat and should be eaten in moderation<br>• Watch how you prepare your eggs, try to use olive oil or low-fat milk for scrambled or fried eggs – even better go for boiled or poached eggs!
L1.3.008	•Go for healthier fats like oily fish, nuts and seeds or unsaturated oils e.g. olive oil
L1.3.009	You are doing really well! Your saturated fat intake is within the recommended levels. It is really important for you to maintain this healthy intake

Salt Decision Tree

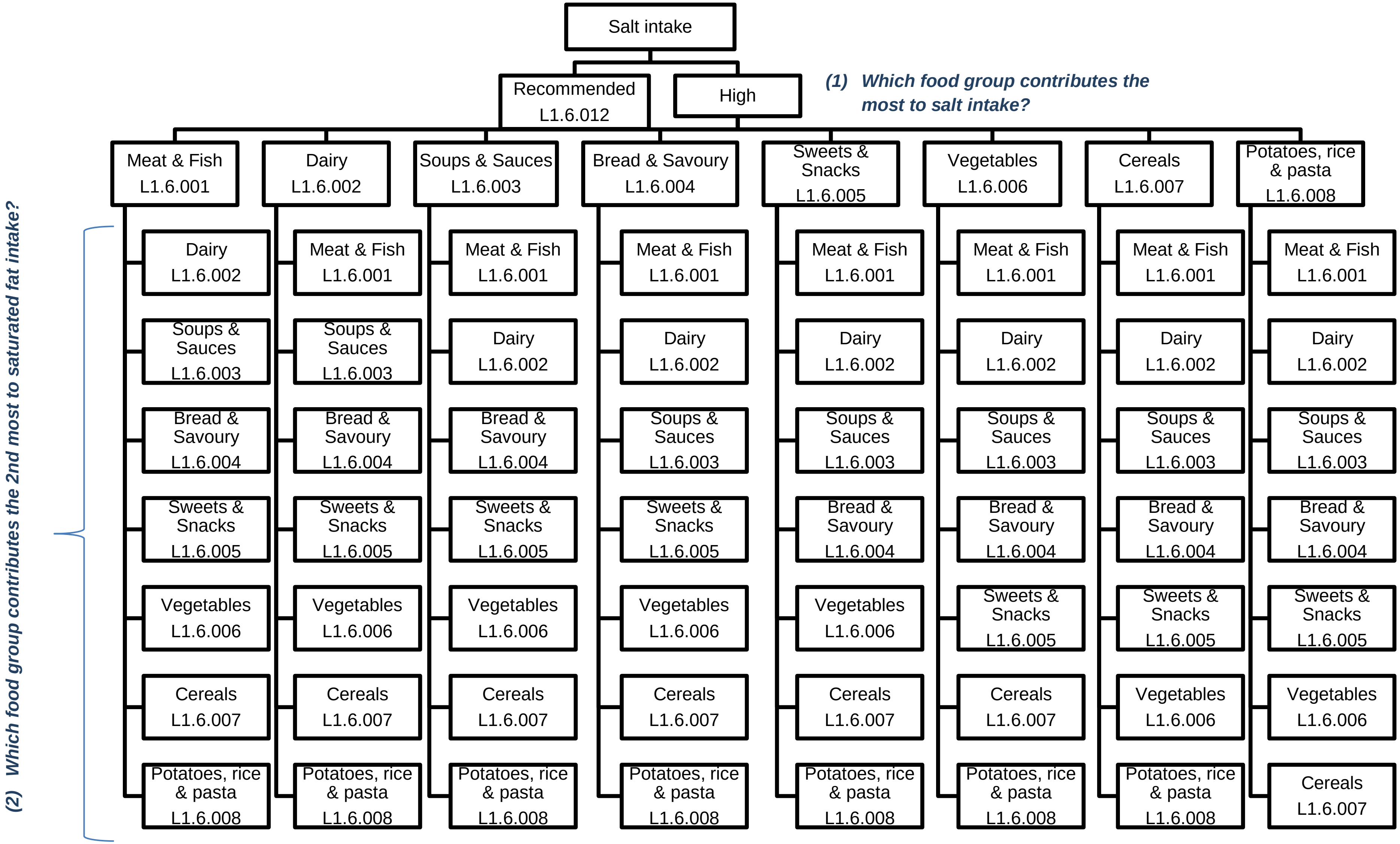

(3) Check whether they add salt when cooking:
If 'No' check whether salt is added at the table. If 'No' give no additional message, if 'Yes' also give message L1.6.009.
If 'Yes' check whether salt is added at the table. If 'No' also give message L1.6.010, or if 'Yes' also give message L1.6.011.

Salt decision tree feedback messages

Message No.	Feedback message
L1.6.001	•Reduce your intake of processed meats and pies; swap salami, ham and bacon for turkey, beef or chicken<br>•Watch out for smoked meats and fish - they are incredibly high in salt
L1.6.002	•Try to consume less cheese; use stronger flavoured cheeses for cooking and sandwiches - you will need a smaller amount
L1.6.003	•Try to choose low-salt or 'reduced salt' products<br>•Next time your shopping try comparing the salt levels in different brands and go for the one with less salt
L1.6.004	•Breads contain a lot of 'hidden' salt<br>•Next time your shopping try comparing the salt levels in different brands and go for the one with less salt
L1.6.005	•Compare the labels of snack foods e.g. crisps and go for ones containing less salt<br>•Watch your portion sizes for nuts and snacks; and swap salted nuts for unsalted nuts
L1.6.006	•Tinned vegetables and baked beans can contain a lot of salt<br>•Go for ones with no added salt or 'low salt' baked beans
L1.6.007	•Try switching to breakfast cereals with no added salt e.g. shredded wholegrain wheat cereals<br>•Compare the salt levels in different brands of cereals and go for the one with less salt; take care with your portion sizes
L1.6.008	•Try to have readymade meals and takeaways only occasionally and watch your portion sizes<br>•Choose pizza's topped with vegetables or chicken instead of pepperoni, bacon or extra cheese<br>•Try to always choose boiled or plain rice instead of egg-fried - it contains more salt
L1.6.009	•Cut back on the amount of salt you add at the table, try to use pepper to season your food instead of salt
L1.6.010	•Reduce the amount of salt you add to foods during cooking - try adding herbs or spices e.g. garlic, lemon, ginger, chilli or black pepper instead of salt
L1.6.011	•Reduce the amount of salt you add to foods during cooking and at the table. When cooking try adding herbs and spices like chilli, lemon, ginger, garlic or black pepper instead
L1.6.012	You are doing really well! Your salt intake is within the recommended levels. It is really important for you to maintain this healthy intake.
