# Supplementary material for: A Dietary Feedback System for the Delivery of Consistent Personalized Dietary Advice in the Web-Based Multicenter Food4Me Study
Source: J Med Internet Res. 2016 Jun 30;18(6):e150. doi: 10.2196/jmir.5620 (PMC4945818; doi:10.2196/jmir.5620)
Supplement: Multimedia Appendix 4 [file jmir_v18i6e150_app4.pdf]

Nutrients and food groups for which personalized feedback was given[a]

**Food groups**	Fruit and vegetables	Information displayed in table showing participants average number of portions compared with guideline amounts
	Wholegrains	
	Dairy products	
	Oily fish	
	Red meat	
**Nutrients[b]**	Protein	Information displayed graphically on a gradation scale showing how the participants intake of each nutrient compares to the Institute of Medicine recommendations (e.g. in recommended range/too high/too low)
	Carbohydrate	
	Total fat	
	Monounsaturated fat	
	Polyunsaturated fat	
	Saturated fat	
	Salt	
	Omega-3	
	Fibre	
	Calcium	
	Iron	
	Vitamin A	
	Folate	
	Thiamin	
	Riboflavin	
	Vitamin B12	
	Vitamin C	

[a] Feedback reports were designed to detail the intake information on the food groups and nutrients listed in the table above

[b] In addition to displaying intake information on each of the 17 nutrients listed in the table above, 3 nutrients were further selected as nutrient-related goals for targeted food based personalized advice
