# Supplementary material for: A Dietary Feedback System for the Delivery of Consistent Personalized Dietary Advice in the Web-Based Multicenter Food4Me Study
Source: J Med Internet Res. 2016 Jun 30;18(6):e150. doi: 10.2196/jmir.5620 (PMC4945818; doi:10.2196/jmir.5620)
Supplement: Multimedia Appendix 5 [file jmir_v18i6e150_app5.pdf]

food4me.org

# PERSONALISED NUTRITION REPORT FOR:

| [Participant ID] | [Participant name] |
|---|---|

## YOUR FOOD4ME NUTRITIONIST:

### REPORT NUMBER: 1

### DATE:

Your personalised nutrition report is based on the nutritional information that you provided for the food4me project, including your dietary questionnaire and your body measurements. Within this report you will find the following information:


A message from your nutritionist
Section 1. How your diet compares to recommendations
Section 2. Your physical characteristics
Section 3. Your nutrient profile
Section 4. Your Personalised Nutrition Advice

## A message from your nutritionist

Dear…………., you are meeting the requirements for the majority of key vitamins and minerals which is great, well done. Your intake of wholegrain foods is lower than recommended and as a result your fibre intake is too. Swapping non-wholegrain cereals for porridge or wholegrain varieties will really help increase your intake of wholegrains and also keep you feeling full for longer. You should try to reduce the number of biscuits you have at each sitting this will help to reduce both your intake of saturated fat and salt which are both higher than recommended, and will help you to lose weight. Increasing your physical activity level will also help to reduce your weight, try going for brisk walks in the evenings. For each section of your report you can access extra information by clicking on the hyperlinks or visiting your homepage on the Food4me website. Good luck!

Outlined below are your main goals to focus on:

•Try to reduce your weight and increase your physical activity
•Try to reduce your intake of salt
•Try to increase your intake of fibre by eating more wholegrain foods
•Try to reduce your intake of saturated fat

We have provided you with tips to help you to achieve these goals in section 4 of your report.

*To go straight to your personalised nutrition advice (section 4), click here*

food4me.org

## Section 1: How your diet compares to recommendations

| Food Group | | Your average number of portions | Guideline amount |
|---|---|---|---|
| Fruit and vegetables | 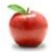 | 5 and ½ a day | At least 5 a day |
| Wholegrains | 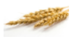 | <50g a day | At least 50g a day |
| Dairy products | 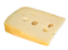 | 1 and ½ a day | 3 a day |
| Oily Fish | 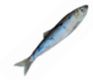 | 2 a week | At least 1 a week |
| Red meat | 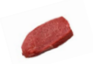 | 3 a week | No more than 3 a week |

*For more information on the food guidelines and general portion sizes click here or visit your personal login page on the Food4me website.*

*To return to the start of your report, click here*

## Section 2: Your Physical Characteristics

Based on the body measurements and physical activity that you recorded, your physical characteristics have been rated below:

Your score

Your height: 1.82m  Your weight: 103kg

| | Underweight <18.5 | Healthy 18.5-24.9 | Overweight 25-29.9 | Obese >30 |
| --- | --- | --- | --- | --- |
| Your BMI: 31.10kg/m$^2$ | | | | |

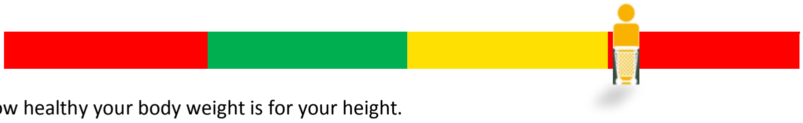

BMI = Body Mass Index. This is an indicator of how healthy your body weight is for your height.

| | Improvement strongly recommended | Improvement recommended | Good, keep up the good work |
| --- | --- | --- | --- |
| Your Physical Activity level: *Based on your physical activity monitor* | | | |

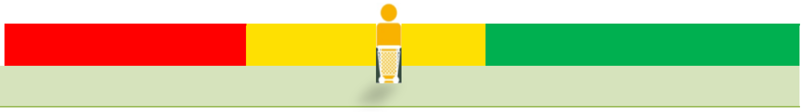

*For more information on these body measurements click here, or for more information on physical activity click here. You can also visit your personal login page on the Food4me website.*

*To return to the start of your report, click here*

## Section 3: Your Nutrient Profile

This section of the report shows your average daily intake for selected macronutrients, fibre, vitamins and minerals in comparison to the Institute of Medicine International recommendations for your age and gender.

- Green: Good, no change recommended
- Yellow: Improvement recommended
- Red: Improvement strongly recommended
- Figure icon: Your intake, you should aim to be in the green

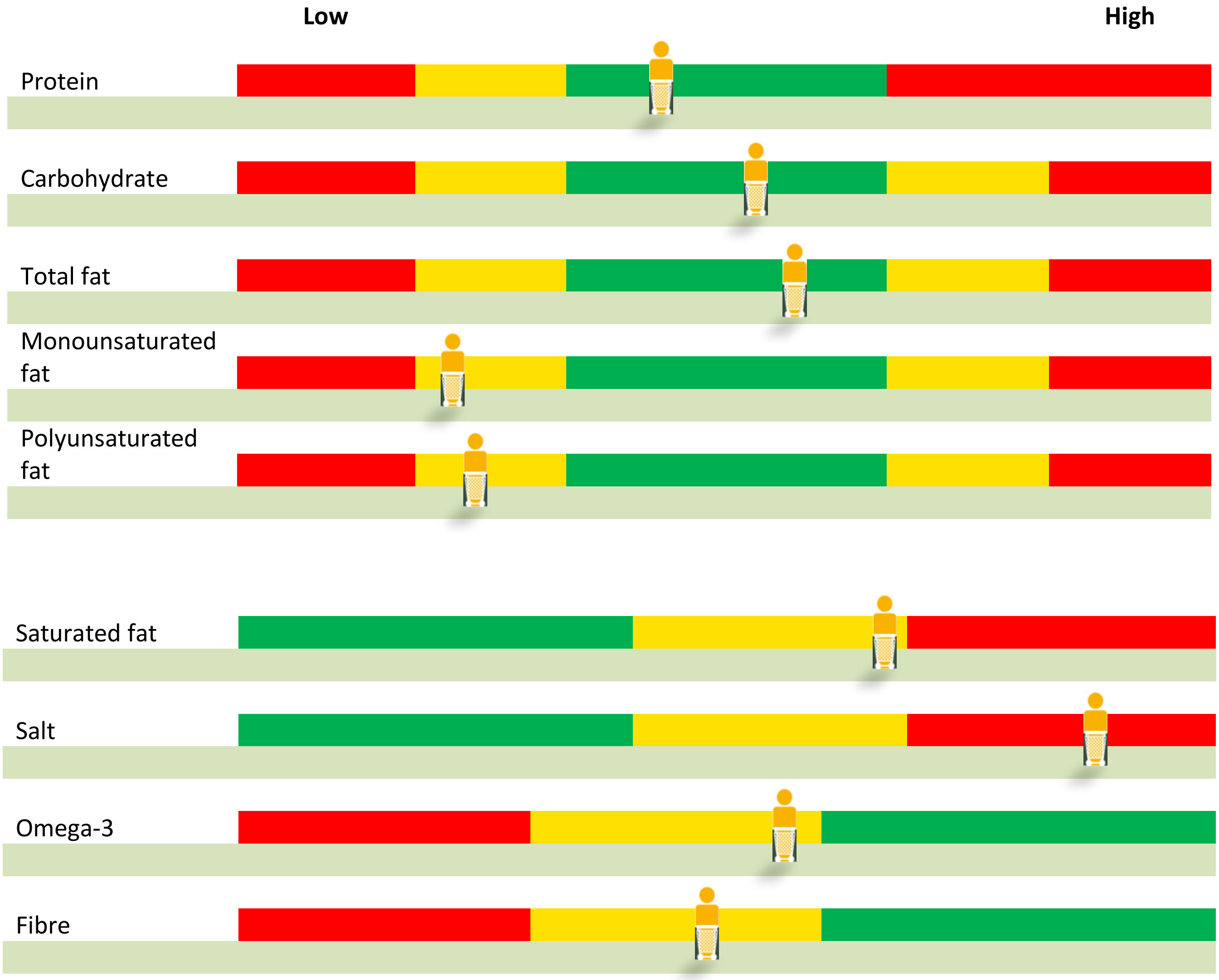

## Your vitamin and mineral intakes

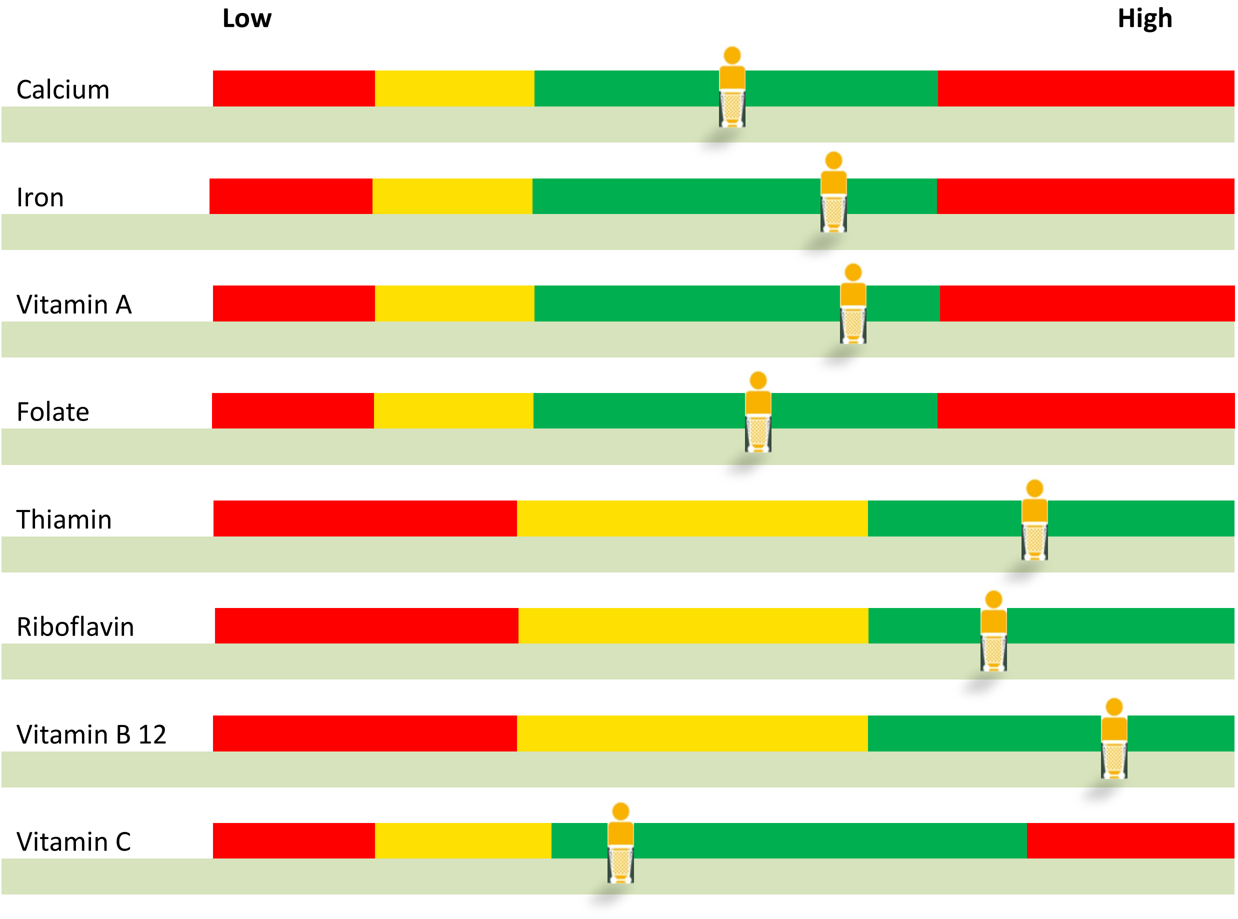


*For more information on any of the nutrients listed above click [here] or check the nutrients section on your personal login page on the Food4me website.*

*To return to the start of your report, click [here]*

# Section 4: Your Personalised Nutrition Advice

## Your Weight and Physical Activity Recommendations

Your BMI is greater than the recommended healthy range, indicating that you are overweight for your height. BMI is a guide that is suitable for estimating an excess or deficit of body fat in the normal population. It does not apply to very muscular individuals because they do not have a typical body composition. We strongly recommend you try to reduce your weight; a weight loss of 0.5-1.0kg (1-2lbs) a week is a realistic goal. Aim to reduce your calorie intake by 500kcal a day. Although you are physically active you are not meeting the daily recommendations; improving your physical activity level will help you to reduce your weight. The following list contains suggestions to help you lose weight:

•Reduce your portion sizes
•Eat regularly and avoid skipping meals
•Reduce your intake of high calorie foods such as high fat snacks and sugary beverages
•Try swapping high fat snacks for fruit
•Choose foods that are low-fat
•Become more physically active, to maintain weight loss 60-90 minutes moderately intense aerobic activities most days a week is recommended. Try brisk walking, swimming or cycling

*For more information on body weight, click here*

## Your dietary goals

As it is very difficult to try to improve all of your nutrient profile at once, we have selected your top 3 nutritional targets to focus on until your next assessment:

| Target | Sources | Goals and Tips |
|---|---|---|
| **Salt** | Smoked and processed foods such as pies, meats, pizza, ready meals and soups<br><br>Your main contributing food groups (and foods):<br>1st **Soups, sauces and spreads** (dark sauces e.g. gravy, stir fry sauces)<br>2nd **Sweets and Snacks** (biscuits) | **How you can reduce your intake of salt:**<br>•Try to choose low-salt or 'reduced salt' products<br>•Next time your shopping try comparing the salt levels in different brands and go for the one with less salt<br>•Compare the labels of snack foods e.g. crisps and go for ones containing less salt<br>•Watch your portion sizes for nuts and snacks; and swap salted nuts for unsalted nuts |

| | | |
|---|---|---|
| **Dietary Fibre** | Fruit and vegetables, wholegrain products including bread, pasta, rice, breakfast cereals, bran, nuts and seeds, oats, barley and pulses e.g. beans, peas and lentils | **<u>How you can increase your fibre intake</u>**:<br>•Well done you are meeting the recommended portions of fruit & veg, try to make sure you are eating a wide variety of fruits and vegetables<br>•Try adding beans to soups, stews and salads and seeds to cereals and yoghurts<br>•Increase your intake of wholegrain breads, crackers and cereals - go for brown rice and wholemeal pasta<br>•Make sure you drink plenty water when increasing your fibre intake<br>•Having a healthy fibre intake has beneficial effects in controlling blood sugar levels |
| | | |
| **Saturated Fat** | Butter and hard margarines, full-fat dairy products, pastries and cakes and processed meats<br><br>Your main contributing food groups (and foods):<br>1st **Sweets and Snacks** (plain biscuits, chocolate biscuits, chocolates)<br>2nd **Meat and Fish** (sausages, burgers, stew) | **<u>How you can reduce your saturated fat intake:</u>**<br>•Reduce your intake of cakes, biscuits and chocolates<br>•Go for treat-size bars and fill up on fruit, vegetables and unsalted nuts<br>•Swap savoury pies and processed meats e.g. burgers, sausages and chicken goujons for lean meats or skinless chicken breast<br>•Trim the fat off meat before cooking it<br>•Go for healthier fats like oily fish, nuts and seeds or unsaturated oils e.g. olive oil |

*For more information on each nutrient, food sources and recommended portion sizes check the nutrients section on your personal login page on the Food4me website.*

*To go directly to the Food4me website click here*

*For more information on alcohol please click here, for advice on smoking please click here*

*To return to the start of your report, click here*

If you have any questions regarding your feedback, please contact the Food4me researchers at food4me@ucd.ie
